# The Role of *CASC2* and *miR-21* Interplay in Glioma Malignancy and Patient Outcome

**DOI:** 10.3390/ijms21217962

**Published:** 2020-10-27

**Authors:** Daina Skiriute, Rytis Stakaitis, Giedrius Steponaitis, Arimantas Tamasauskas, Paulina Vaitkiene

**Affiliations:** 1 Laboratory of Molecular Neurooncology, Neuroscience Institute, Lithuanian University of Health Sciences, Eiveniu str. 4, LT-50161 Kaunas, Lithuania; rytis.stakaitis@lsmuni.lt (R.S.); giedrius.steponaitis@lsmuni.lt (G.S.); arimantas.tamasauskas@kaunoklinikos.lt (A.T.); 2 Laboratory of Molecular Neurobiology, Neuroscience Institute, Lithuanian University of Health Sciences, Eiveniu str. 4, LT-50161 Kaunas, Lithuania; paulina.vaitkiene@lsmuni.lt

**Keywords:** *CASC2*, *miR-21*, glioma, *IDH1* status, patient survival

## Abstract

Recently long non-coding RNAs (lncRNAs) were highlighted for their regulatory role in tumor biology. The novel human lncRNA cancer susceptibility candidate 2 (*CASC2*) has been characterized as a potential tumor suppressor in several tumor types. However, the roles of *CASC2* and its interplay with *miR-21* in different malignancy grade patient gliomas remain unexplored. Here we screened 99 different malignancy grade astrocytomas for *CASC2*, and *miR-21* gene expression by real-time quantitative polymerase chain reaction (RT-qPCR) in isocitrate dehydrogenase 1 (*IDH1*) and O-6-methylguanine methyltransferase (*MGMT*) assessed gliomas. *CASC2* expression was significantly downregulated in glioblastomas (*p* = 0.0003). Gliomas with low *CASC2* expression exhibited a high level of *miR-21*, which was highly associated with the higher glioma grade (*p* = 0.0001), *IDH1 wild type* gliomas (*p* < 0.0001), and poor patient survival (*p* < 0.001). Taken together, these observations suggest that *CASC2* acts as a tumor suppressor and potentially as a competing endogenous RNA (ceRNA) for *miR-21,* plays important role in *IDH1 wild type* glioma pathogenesis and patients’ outcomes.

## 1. Introduction

Malignant gliomas, especially glioblastomas, are highly infiltrative, rapidly growing, aggressive, heterogeneous, chemo-resistant, and lethal neoplasms [1]. The accurate distinction between the different malignancy types has significant prognostic and therapeutic implications [2]. A thorough study of the molecular mechanisms of the formation and progression of glioma is essential for the screening of valuable diagnostic and prognostic molecular markers. Long non-coding RNAs (lncRNAs) were first recognized as being crucial regulators of gene expression in a wide range of biological context, including cancer [3]. Various lncRNAs, including homeobox (*HOX*) transcript antisense RNA (*HOTAIR*), metastasis associated lung adenocarcinoma transcript *(MALAT*), colorectal neoplasia differentially expressed (*CRNDE*), have been identified as novel players in glioma pathogenesis demonstrating associations with tumor subtype, histological stage, tumor isocitrate dehydrogenase (*IDH*) mutational status, chemosensitivity, and patient survival [4,5,6,7,8].

Steadily growing evidence on the ability of lncRNAs to interact with DNA, RNA, and proteins acting as tethers, guides, decoys, and scaffolds, includes them in the posttranscriptional regulatory network in cancer biology [9]. Moreover, the increasing evidence suggests an interplay between microRNAs and lncRNAs [10,11]. A large number of lncRNAs act as a competing endogenous RNAs (ceRNA) or sponges for microRNAs, for example, phosphatase and tensin homolog pseudogene 1 (*PTENpg1)*, *HOTAIR* [11,12]. The lncRNA gene cancer susceptibility gene 2 (*CASC2*) has been characterized as tumor suppressor in various human malignancies [13,14,15,16,17,18,19]. Although the deregulated expression of *CASC2* in cancer enhances its tumorigenic properties, however, the literature evidence limits current knowledge on the pathophysiological implications and the roles of *CASC2*, and its interplay with *miR-21* in the pathology of gliomas [20].

In this study, we assessed levels of *CASC2* and *miR-21* and their interplay in different grades of glioma. Our findings indicate that *CASC2* was proportionally downregulated in progressed gliomas, while *miR-21* expression was inversely associated with *CASC2* expression, malignancy grade, and patient survival. Here we demonstrate *CASC2* acting as a tumor suppressor and likely interacting with *miR-21* in *IDH1* wild type gliomas.

## 2. Results

### 2.1. *CASC2* and *miR-21* Associations with Patient Clinical Parameters

To evaluate whether *CASC2* and *miR-21* were associated with glioma patient clinical parameters, we divided the samples into “low” and “high” (below and above the gene’s mean expression of all samples, respectively) gene expression groups. The threshold for “low” and “high” expression group of *CASC2* and *miR-21* was −5.865 and 3.448 (log_2_^(2^−∆Ct)^), respectively. As shown in Table 1, lower *CASC2* and higher *miR-21* expression were observed more frequently in patients with advanced tumor stage (IV grade gliomas/glioblastomas) (*p* < 0.0001). Furthermore, *IDH1* wild-type gliomas more frequently had lower *CASC2* and higher *miR-21* expression (*p* = 0.037 and *p* < 0.0001, respectively). 

### 2.2. *CASC2* and *miR-21* Expression in High Grade and IDH1wt Gliomas

**Figure 1:**
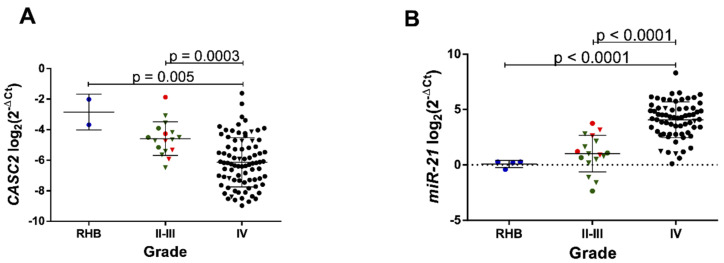
Cancer susceptibility gene 2 (*CASC2*) and *miR-21* gene expression are associated with glioma malignancy grade. (**A**) *CASC2* expression measured by RT-qPCR in RHB (reference human brain, *n* = 2), II-III malignancy grade gliomas (*n* = 17) and IV grade gliomas, *n* = 82 (glioblastomas). (**B**) *miR-21* expression measured in the same patient postoperative tumor tissue by RT-qPCR in RHB (*n* = 4), II-III (*n* = 17) and IV grade (*n* = 66) gliomas. The lines in the graphs indicate mean with the SD. Color corresponds to different glioma malignancy grade: green—grade II, red—grade III, black—grade IV gliomas. Triangle shape corresponds to isocitrate dehydrogenase 1 mutated C. 395G > A (*IDH1mut*) glioma, circle shape—isocitrate dehydrogenase 1 wild-type (*IDH1wt*).

**Figure 2:**
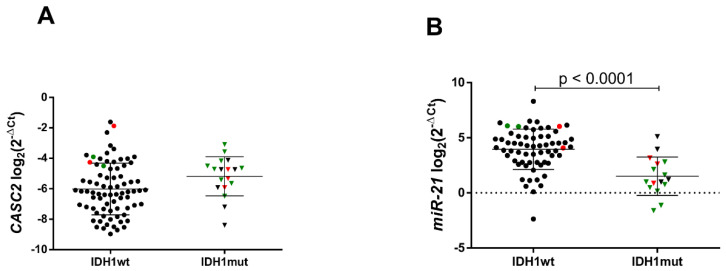
*CASC2* (**A**) and *miR-21* (**B**) gene expression in *IDH1wt* and *IDH1mut* gliomas. The lines in the graphs indicate mean with the SD. Color corresponds to different glioma malignancy grade: green reflects grade II, red—grade III, black—grade IV gliomas. Triangle shape corresponds to isocitrate dehydrogenase 1 mutated C. 395G > A (*IDH1mut*) glioma, circle shape—isocitrate dehydrogenase 1 wild-type (*IDH1wt*).

Whether the activity of *CASC2* and *miR-21* was linked to the clinical progression of gliomas, we examined gene expression in grade II–III and IV gliomas. Here we show significant *CASC2* expression loss and drastic rise of *miR-21* expression in glioblastomas as compared to the average expression levels in lower grade (II–III) gliomas (*p* = 0.0003 and *p* < 0.0001, respectively) and control non-cancerous brain tissues (*p* = 0.005 and *p* < 0.0001, respectively) (Figure 1A,B). When all samples were divided into *IDH1* gene mutated (*IDH1mut*, *n* = 18) and *IDH1* wild-type (*IDH1wt*, *n* = 78) gliomas, we observed a tendency of lower expression of *CASC2* in *IDH1wt* (*p* = 0.053) and highly significant relationship between higher *miR-21* expression and *IDH1wt* gliomas (*p* < 0.0001) (Figure 2A,B). 

### 2.3. *CASC2* and *miR-21* Interplay in Gliomas

**Figure 3:**
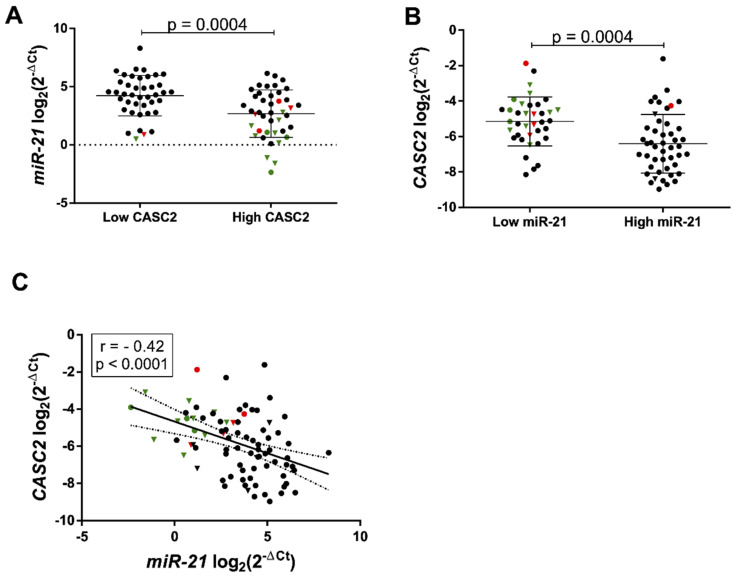
*CASC2* and *miR-21* interplay in gliomas. (**A**) *miR-21* gene expression in low (*n* = 41) and high (*n* = 42) *CASC2* expression groups in all patient gliomas. (**B**) *CASC2* gene expression in low (*n* = 37) and high (*n* = 46) *miR-21* gene expression groups in all patient gliomas. (**C**) Expression correlation between *CASC2* and *miR-21* in gliomas (*r* = −0.42, *p* < 0.0001, *n* = 83) visualized as a scatter plot. The lines in the graphs indicate mean with the SD. Color corresponds to different glioma malignancy grade: green reflects grade II, red—grade III, black—grade IV gliomas. Triangle shape corresponds to *IDH1mut* glioma, circle shape—*IDH1wt*.

Several reports have suggested that lncRNAs may function as a molecular sponge or competing endogenous RNA in modulating miRNAs, suggesting that it could be an inverse correlation between lncRNA and miRNAs [21]. It was shown that *miR-21* can bind to *CASC2* directly by the putative miRNA response element (MRE) [20]. Here at the clinical level, we further confirm the recently reported interaction between *CASC2* and *miR-21* in glioma cell lines. We show that significantly higher *miR-21* gene expression was observed in the “low” *CASC2* group as compared to “high” and vice versa (*p* = 0.0004, Figure 3A,B). Correlation analysis revealed moderate negative association between *CASC2* and *miR-21* expression in gliomas (*r*^2^ = −0.42, *n* = 83, *p* < 0.0001, Figure 3C). 

### 2.4. Survival Analysis

**Figure 4:**
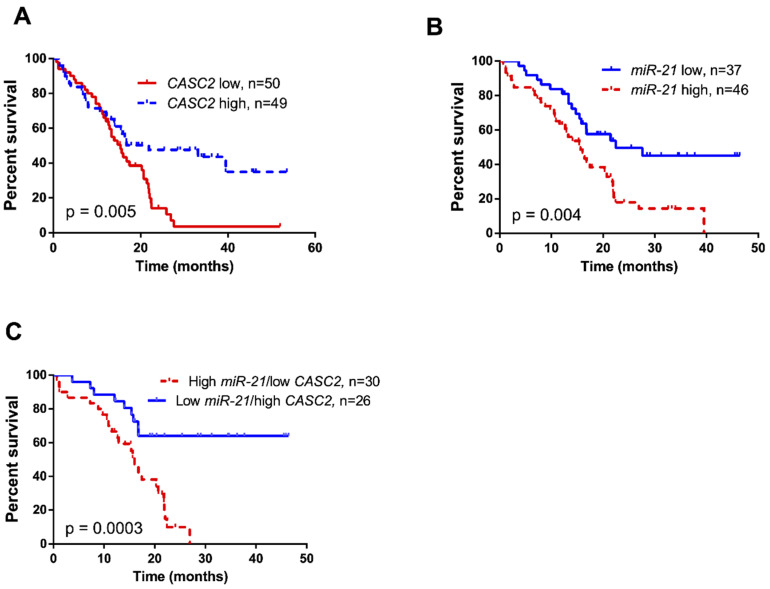
Kaplan–Meier curves for glioma patient survival correlation with (**A**) *CASC2* expression (Log-rank test, χ^2^ = 7.777, df = 1, *p* = 0.005), (**B**) *miR-21* expression (Log-rank test, χ^2^ = 8.518, df = 1, *p* = 0.004), and (**C**) combined *miR-21* and *CASC2* expression (Log-rank test, χ^2^ = 12.91, df = 1, *p* = 0.0003) in glioma tumor tissue.

Kaplan–Meier survival analysis showed highly significant association between low *CASC2* expression levels (*n* = 50) and worse patient outcome (Log-rank test, χ^2^ = 7.777, df = 1, *p* = 0.0053; Figure 4A), while patients with low *miR-21* expression (*n* = 37) showed significantly increased overall survival, compared to patients with high *miR-21* expression (*n* = 46) (Log-rank test, χ2 = 8.518, df = 1, *p* = 0.0035; Figure 4B). The combined effect of low *CASC2* and high *miR-21* expression (*n* = 30) in glioma was shown to be associated with significantly decreased overall survival compared to patients with the combination of high *CASC2* and low *miR-21* expression (*n* = 26) in tumor tissue (Log-rank test, χ^2^ = 12.91, df = 1, *p* = 0.0003; Figure 4C). Univariate Cox regression model revealed that patients’ clinical characteristics such as age and tumor stage, *IDH1* status was associated with their survival as well as *CASC2, miR-21*, and combined *CASC2/miR-21* expression. However, multivariate analysis showed that only patient age and tumor stage were covariates associated with the overall survival of glioma patients (Table 2). 

## 3. Discussion

The key finding of the current study is that downregulation of lncRNA *CASC2* and upregulation of *miR-21* expression is associated with glioma progression. Our results show that *CASC2* downregulation is associated with highly expressed *miR-21* and poor patient outcome. Moreover, here we show that highly active *CASC2* significantly might have suppressed *miR-21* levels in *IDH1* wild-type gliomas. 

Current knowledge on the involvement and function of lncRNA *CASC2* in glioma evidences the availability of a small amount of data from clinical samples. Downregulation of *CASC2* in glioma tissue was showed by Wang et al. [20] in a limited sample of 24 patients, while Liao et al. revealed *CASC2* playing a role in modulating glioma temozolomide (TMZ) chemoresistance in 57 patient samples [22]. In agreement with our data, *CASC2* expression in both studies was shown to correlate with glioma malignancy grade inversely. Recently, several studies including patient samples have been carried out on *CASC2* expression in other malignancies. In particular, *CASC2* acts as a tumor suppressor in endometrial, colorectal, lung, stomach, renal, gastric cancers, and osteosarcomas [13,14,15,16,17,18,19]. In NSCLC (non-small-cell lung carcinoma) patients (*n* = 76), *CASC2* expression was downregulated proportionally to the pathological stage and associated with tumor size, and this gene was an independent predictor for overall survival [13]. In 76% CRC (colorectal cancer) patients (*n* = 68) *CASC2* low expression was associated with tumor stage [14]. In RCC (renal cell carcinoma) (*n* = 32), *CASC2* was significantly downregulated compared with the matched normal tissue [15]. In gastric cancer tissue (*n* = 67) and cell lines, *CASC2* expression was downregulated [17] and low *CASC2* level in tissue correlated with the vessel invasion, tumor stage, metastasis, and poor patient survival [19]. In osteosarcoma, *CASC2* expression downregulation was observed in patient tissue samples and cell lines, and low expression in tissue was associated with poor tumor differentiation, higher malignancy grade, and shortened patient survival [18]. To sum up, recent scientific work and our research in astrocytic gliomas support evidence that *CASC2* gene expression is downregulated proportionally to tumor stage, indicating the suppressive role of *CASC2* in malignancy progression. Functional studies in vitro in various cancer cell lines confirmed *CASC2* acting as a tumor suppressor as when overexpressed *CASC2* was able to inhibit cell proliferation, cell growth, migration and invasion, and to induce apoptosis [20]. 

Emerging evidence revealed a new mechanistic role of lncRNAs as part of a posttranscriptional regulatory network in cancer biology. Recent data suggest that coding and non-coding RNAs can regulate one another through their ability to compete for miRNA binding through typical MREs (miRNA response elements). LncRNAs can act as competing endogenous RNAs (ceRNA) or miRNA “sponges”, which can sequester miRNAs, therefore preventing single or multiple miRNA from binding to their proper target RNAs and protecting them from suppression [23]. Importantly, micro RNAs also regulate lncRNAs [10]. Recently, it was demonstrated that *CASC2* in colorectal cancer is functioning as ceRNA for *miR-18a*, thereby modulating the expression of target gene *PIAS3*, and subsequently inhibiting CRC cell proliferation and tumor growth [14]. Studies in hepatocellular carcinoma revealed that *CASC2* prohibited mesenchymal–epithelial transition progression and exerted anti-metastatic effect via *CASC2/mirR-396/FBXW7* axis [9]. Wang and colleagues demonstrated *CASC2* and *mir-21* reciprocal interaction in glioma cell lines U251 and U87 [20]. Similarly, Liao et al. [22] study showed *CASC2* interaction with *miR-181a* and *PTEN* gene in regulating chemosensitivity in temozolomide resistant glioma cells. However, as to our knowledge, no studies are demonstrating *CASC2* and *miR-21* interaction in patient glioma samples and evaluating its clinical relevance.

Consistent with published reports on *CASC2/mir-21* interaction in glioma and non-small cell lung cancer cells in vitro [20,24], we provide evidence in patient gliomas that *CASC2* and *miR-21* play antagonistic roles and potentially interact in glioma progression. In support of this, the RT-qPCR analysis showed that *miR-21* expression is moderately upregulated in low-grade astrocytoma and even highly upregulated in malignant glioblastoma, while high expression of *CASC2* in tumors might be responsible for the decrease of *miR-21* expression. Agreeing with our findings, *miR-21* has been well studied in gliomas with particularly high expression. *miR-21* is consistently upregulated in astrocytic tumors (grade II–IV) and downmodulates an entire set of oncosuppressor genes, for example, *PTEN* [25]. High *miR-21* expression in tumor tissue was highly associated with aggressive clinicopathological features and poor overall patient survival (*n* = 152) [26]. In the current study, we found a correlation between high *mir-21* expression and *IDH1wt* gliomas. It is known that mutation in isocitrate dehydrogenase 1 (*IDH1mut*) is associated with distinct glioma cell metabolic profile, hypermethylated phenotype, and significantly longer overall survival as compared to patients with *IDH1wt* [27,28]. Our results further indicate *miR-21* predictive value in *IDH1wt* associated gliomagenesis. 

In summary, lncRNA *CASC2* was found as a tumor suppressor and downregulated in low-grade astrocytomas and highly malignant glioblastomas as compared to healthy brain tissue. *miR-21* was inversely expressed with *CASC2* in gliomas and correlated with *IDH1wt* glioma and poor patient prognosis.

## 4. Material and Methods

### 4.1. Ethics

The research was reviewed and approved by the Kaunas Regional Bioethics Committee (protocol: L6.1-07/09, permission code: P2-9/2003, date: 10 October 2010) and performed following the Lithuanian regulations alongside with the principles of the Helsinki and Taipei Declarations [29,30]. 

### 4.2. Patient Sample

Due to rare occurrence of the disease, the maximum possible number of samples were included into the study. A total of 99 samples of different malignancy grade astrocytomas were analyzed for *CASC2* and *miR-21* expression: 17 grade II–III astrocytic gliomas and 82 grade IV astrocytic gliomas/glioblastomas. *CASC2* expression was analyzed in 99 samples, while *miR-21* in 83 samples. *IDH1* status was obtained for 96 patients, *MGMT* promoter methylation status was determined for 89 samples. Tissue samples were prospectively collected at the Department of Neurosurgery of Lithuanian University of Health Sciences, during the period of 2015–2018. The pathological review was performed on each sample to confirm the diagnosis of astrocytic glioma. All tissue samples were stored in liquid. None of the patients had received preoperative chemotherapy or radiotherapy. All patients signed written consent forms. Overall survival was calculated from the day of surgery to the death or last follow-up.

### 4.3. RNA and DNA Extraction

Total and small RNAs (<200 nt) were extracted from 30–40 mg snap-frozen (−196 °C) post-surgical tumor samples applying cryogenic mechanical grinding, ultrasonic homogenization at 20% amplitude for 1 s on/off pulsation and using mirVana™ miRNA Isolation Kit (Thermo Fisher Scientific, Carlsbad, CA, USA). Procedures were done according to the manufacturer’s instructions. The RNA concentration was determined using a NanoDrop 2000 (Thermo Fisher Scientific, Wilmington, DE, USA). Quality of extracted small RNAs was evaluated with a Small RNA analysis kit (Agilent, Santa Clara, CA, USA) on a 2100 Bioanalyzer (Agilent, Santa Clara, CA, USA). 

DNA was extracted from ≈40 mg frozen tumor tissue using the desalting method with chloroform, and Proteinase K. DNA concentration was measured with a NanoDrop 2000 system.

### 4.4. *CASC2* Gene Expression Analysis

cDNA synthesis was performed using 2 µg of RNA, hexamer primers, “Multiscribe™ Reverse Transcriptase” reverse transcriptase, and according to the manufacturer’s recommendations, using “High-Capacity cDNA Reverse Transcription Kit” (Applied Biosystems, Foster City, CA, USA). RT-qPCR was conducted using a “AB 7500 Fast Real-time PCR system” (Applied Biosystems, Foster City, CA, USA). *CASC2* gene primer sequences were as follows: forward 5‘-GCACATTGGACGGTGTTTCC-3’; reverse 5’-CCCAGTCCTTCACAGGTCAC-3’ [31]. All amplification reactions were performed in 96-well plates and each sample was tested in three replicates. For normalization, the geometric average of five housekeeping genes’ expression glyceraldehyde 3-phosphate dehydrogenase (*GAPDH*), tyrosine 3-monooxygenase/tryptophan 5-monooxygenase activation protein zeta (*YWHAZ*), *β-actin*, 18S ribosomal RNA (*18s rRNA*), hypoxanthine phosphoribosyltransferase 1 *(HPRT1*) was used. As endogenous control “FirstChoice Human Brain Reference Total RNA” (RHB) (Ambion, Austin, TX, USA) was used. In order to quantify samples in 95% of the cases, samples with a standard deviation of more than 0.25 were eliminated from the analysis. Gene expression was calculated as 2^−∆Ct^ values and in figures presented as log-transformed values.

### 4.5. *miR-21* Gene Expression Analysis

In total, 10 ng of purified micro RNAs was synthesized to cDNA using “TaqMan Advanced miRNA cDNA Synthesis Kit” (Thermo Fisher Scientific, Pleasanton, CA, USA). Expression profile of mature micro RNA 21 was detected performing RT-qPCR on “7500 Fast Real-Time PCR system” (Applied Biosystems, Foster City, CA, USA) in three replicates using “TaqMan Fast Advanced Master Mix” (Thermo Fisher Scientific, Austin, TX, USA) and *hsa-miR-21-5p* probes (Assay ID: 477975_mir). In addition, *hsa-miR-191-5p* (Assay ID: 477952_mir) and *hsa-miR-361-5p* (Assay ID: 478056_mir) were measured in order to normalize the data. Relative quantitation of *hsa-miR-21-5p* expression for each sample was calculated according to Equation (1):

(1) $$∆Ct_{miR21}=\;Ct_{miR21}-\;\sqrt[{2}]{Ct_{miR191}\times Ct_{miR361}}\;\mathrm{and}\;2^{-∆Ct\mathrm{miR}-21}.$$

### 4.6. *IDH1* Mutation Detection

The most common *IDH1* gene mutation R132H in gliomas was analyzed in all the specimens applying custom TaqMan single nucleotide polymorphism (SNP) genotyping assays. PCR was carried out in a total volume of 12 µL consisting of “TaqMan™ Universal Master Mix II” (Thermofisher Scientific, Carlsbad, CA, USA), TaqMan probes and 20 ng of purified tumor DNA. All the procedures were accomplished according to the manufacturer’s recommendations. Fluorescence was measured with “7500 Fast Real-Time PCR System” (Applied Biosystems, Foster City, CA, USA). Amplification of DNA with Wild or Mutant allele labelled with VIC or FAM dyes indicated different gene variants, respectively.

### 4.7. *MGMT* Methylation Detection

*MGMT* promoter methylation status was determined using methylation-specific PCR (MSP). The reaction performed in 15 µL of total volume consisted of 7.5 µL “Hot Start PCR Master Mix” (Thermofisher Scientific, Carlsbad, CA, USA); 4.5 µL nuclease-free water (Thermofisher Scientific, Carlsbad, CA, USA); 1 µL (10 pmol/µL) of each primer, specific to methylated/unmethylated promoter; ≈20 ng of bisulfite-treated DNA as a template. Primer sequences for methylated *MGMT* sequence were 5’-GGACGTTAAGGGTTTAGAGC-3’ (sense), 5’-CAATACACGACCTCGTCAC-3’ (antisense), for unmethylated—5’-GGATGTTAAGGGTTTAGAGT-3’ (sense), 5’-CAATACACAACCTCATCAC-3’ (antisense). In addition, three controls were performed: positive—“Bisulfite converted Universal Methylated Human DNA Standard and Control primer” (ZymoResearch, Irvine, CA, USA); negative—bisulfite treated human blood lymphocytes DNA; water control. PCR products were visualized using agarose gel electrophoresis. Each sample methylation status was evaluated according to visible signals and documented using 0 (unmethylated) and 1 (methylated) system.

### 4.8. Statistical Analysis

Statistical analysis was performed using GraphPad Prism version 6.0 (San Diego, CA, USA). Continuous variables were checked for normal distribution using a Shapiro–Wilk statistics and compared by a Student’s *t*-test when normally distributed or by a Mann–Whitney U test when data distributed non-normally. Pearson’s correlation coefficient was calculated to test the association between different gene expression. Pearson’s chi-squared test was used for comparison of categorical variables. Kaplan–Meier curves were compared using a Log-rank analysis in different gene expression groups. For regression analysis, gene expression values were categorized as “low” or “high” according to a log-transformed gene expression values which were above or below all sample expression means, respectively. The significance level was considered when *p*-value < 0.05 (*).

## Figures and Tables

**Table 1:** The relationship between cancer susceptibility gene 2 (*CASC2*) and *miR-21* gene expression in glioma tissue and patient clinical characteristics. Pearson’s χ^2^-test was used for comparison of categorical variables.

Variable	Total No	*CASC2* Expression			Total No	*miR-21* Expression		
		Low (%)	High (%)	*p*-Value		Low (%)	High (%)	*p*-Value
Gender								
Male	45	25 (55.6)	20 (44.4)	0.422	37	13 (35.1)	24 (64.9)	0.182
Female	54	25 (46.3)	29 (53.7)		46	24 (52.2)	22 (47.8)	
Age, yr								
<56	47	23 (48.9)	24 (51.1)	0.841	42	24 (57.1)	18 (42.9)	0.027
≥56	52	27 (51.9)	25 (48.1)		41	13 (31.7)	28 (68.3)	
Grade								
II-III	17	2 (11.8)	15 (88.2)	<0.0001	17	16 (94.1)	1 (5.9)	<0.0001
IV	82	48 (58.5)	34 (41.5)		66	21 (31.8)	45 (68.2)	
*IDH1*								
Wt	78	44 (56.4)	34 (43.6)	0.037	64	21 (32.8)	43 (67.2)	<0.0001
Mut	18	5 (27.8)	13 (72.2)		16	14 (87.5)	2 (12.5)	
*MGMT*								
Unmeth	48	25 (52.1)	23 (47.9)	1	40	16 (40)	24 (60)	0.812
Meth	41	22 (53.7)	19 (46.3)		33	15 (45.5)	18 (54.5)	
*miR-21*								
low	37	11 (29.7)	26 (70.3)	0.002				
high	46	30 (62.2)	16 (34.8)					

**Table 2:** Cox regression analysis of different clinicopathological variables, *CASC2* and *miR-21* expression.

Characteristics	Univariate Analysis		Multivariate Analysis	
	HR (95% CI)	*p*-Value	HR (95% CI)	*p*-Value
Age (<56 vs. ≥56)	0.216 (0.123–0.381)	<0.0001	0.408 (0.215–0.775)	0.006
Gender (female vs. male)	0.868 (0.537–1.404)	0.564	NA	
Tumor grade (II–III vs. IV)	0.069 (0.017–0.284)	<0.0001	0.100 (0.019–0.526)	0.007
*IDH1^R132H^*	0.160 (0.064–0.404)	<0.0001	0.809 (0.244–2.682)	0.729
*MGMT* (methylated vs. non methylated)	0.722 (0.435–1.199)	0.208	NA	
*CASC2*	0.497 (0.301–0.821)	0.006	0.751 (0.389–1.450)	0.393
*miR-21*	2.285 (1.290–4.045)	0.005	1.173 (0.584–2.358)	0.653
*CASC2* high/*miR-21* low	0.259 (0.118–0.570)	0.001	NA	
